# Broadband All-angle Negative Refraction by Optimized Phononic Crystals

**DOI:** 10.1038/s41598-017-07914-1

**Published:** 2017-08-07

**Authors:** Yang Fan Li, Fei Meng, Shiwei Zhou, Ming-Hui Lu, Xiaodong Huang

**Affiliations:** 1 Faculty of Science, Engineering and Technology, Swinburne University of Technology, Hawthorn, Victoria 3122 Australia; 2 Centre for Innovative Structures and Materials, School of Engineering, RMIT University, Melbourne, 3001 Australia; 3 National Laboratory of Solid State Microstructures and Department of Materials Science and Engineering, Nanjing University, Nanjing, 210093 People’s Republic of China; 4 Key Laboratory of Advanced Technology for Vehicle Body Design and Manufacture, Hunan University, Changsha, 410082 People’s Republic of China

## Abstract

All-angle negative refraction (AANR) of phononic crystals and its frequency range are dependent on mechanical properties of constituent materials and their spatial distribution. So far, it is impossible to achieve the maximum operation frequency range of AANR theoretically. In this paper, we will present a numerical approach for designing a two-dimensional phononic crystal with broadband AANR without negative index. Through analyzing the mechanism of AANR, a topology optimization problem aiming at broadband AANR is established and solved by bi-directional evolutionary structural optimization method. The optimal steel/air phononic crystal exhibits a record AANR range over 20% and its refractive properties and focusing effects are further investigated. The results demonstrate the multifunctionality of a flat phononic slab including superlensing effect near upper AANR frequencies and self-collimation at lower AANR frequencies.

## Introduction

Since Veselago^1^ first predicted the left-hand material (LHM) with simultaneously negative permittivity and permeability, artificial materials with negative refraction have attracted considerable attention due to their interesting and unusual behaviors. By virtue of double negative effective index, super resolution beyond the traditional diffraction limit was first achieved for electromagnetic waves by Pendry with a flat optical lens, called a superlens^2^. On the other hand, negative refraction of electromagnetic waves can also result from Bragg scattering effects of photonic crystals, where the effective index is still positive^3, 4^. Similar to the perfect lens consisting of LHMs, negative refraction in photonic crystals can also lead to a flat superlens^5, 6^.

Sparked by these pioneering works on electromagnetic waves, increasing efforts have been devoted to exploring similar phenomena in phononic crystals (PnCs) and acoustic metamaterials theoretically and experimentally, such as achieving negative refraction^7–19^ and reflection^20^ of acoustic waves, and very recently realizing acoustic topological insulators^21–23^. These explorations enable us to manipulate the propagation of acoustic waves better and are promising for engineering devices with unconventional functions, for example, focusing of sound waves with super resolution. Compared with acoustic metamaterials whose lattice constant is unusually two orders shorter than the relevant wavelength, PnCs are more preferable to achieve subwavelength focusing at the ultrasound regime as their lattice constant is comparable to the wavelength. There are two different approaches to realize negative refraction in PnCs. One is exploiting the natural opposite direction of wave vector and energy flow at high-order band, typically at the second band, which has been extensively discussed in the literature^10, 11, 13, 15, 16^. This mechanism comes along with a backward-wave effect and the negative effective index, and is similar to the negative refraction by metamaterials. The other is achieving negative refraction at the first phononic band by intensive scattering of constituents, resulting in positive effective properties and PnCs that behave like uniform right-handed materials (RHMs)^8, 9^. Such designs have advantages of single mode, high transmission and possible all-angle negative refraction (AANR), which is of particular interest in this paper. To our knowledge, AANR at the first band was only reported in a 2D square lattice of mercury/water system with a relative AANR frequency range about 6% around frequency 0.24 * 2*πc*_*water*_/*a* ^8^.

A critical question is how to attain the maximum operation frequency range of AANR so as to realize its full potential. AANR in PnCs is essentially a band structure controlled behavior, thus influenced by mechanical properties of the constituent materials, and their spatial distributions (or called structure) within the primitive unit cell^24–27^. Due to the infinite possibility of material distribution, it is impossible to obtain the best structure for AANR from a physical point of view, yet it is anticipated to answer this question by a multidisciplinary approach. In this paper, we establish a numerical approach to achieve AANR behavior in PnCs by integrating the underlying physical mechanism into a topology optimization problem. To seek the best configuration of PnCs with broadband AANR, we employ the bi-directional evolutionary structural optimization (BESO) method which has been proven to be very effective and robust in the design of photonic/phononic band gap crystals, as well as photonic structures with AANR^28–32^.

## Results

### AANR mechanism

**Figure 1:**
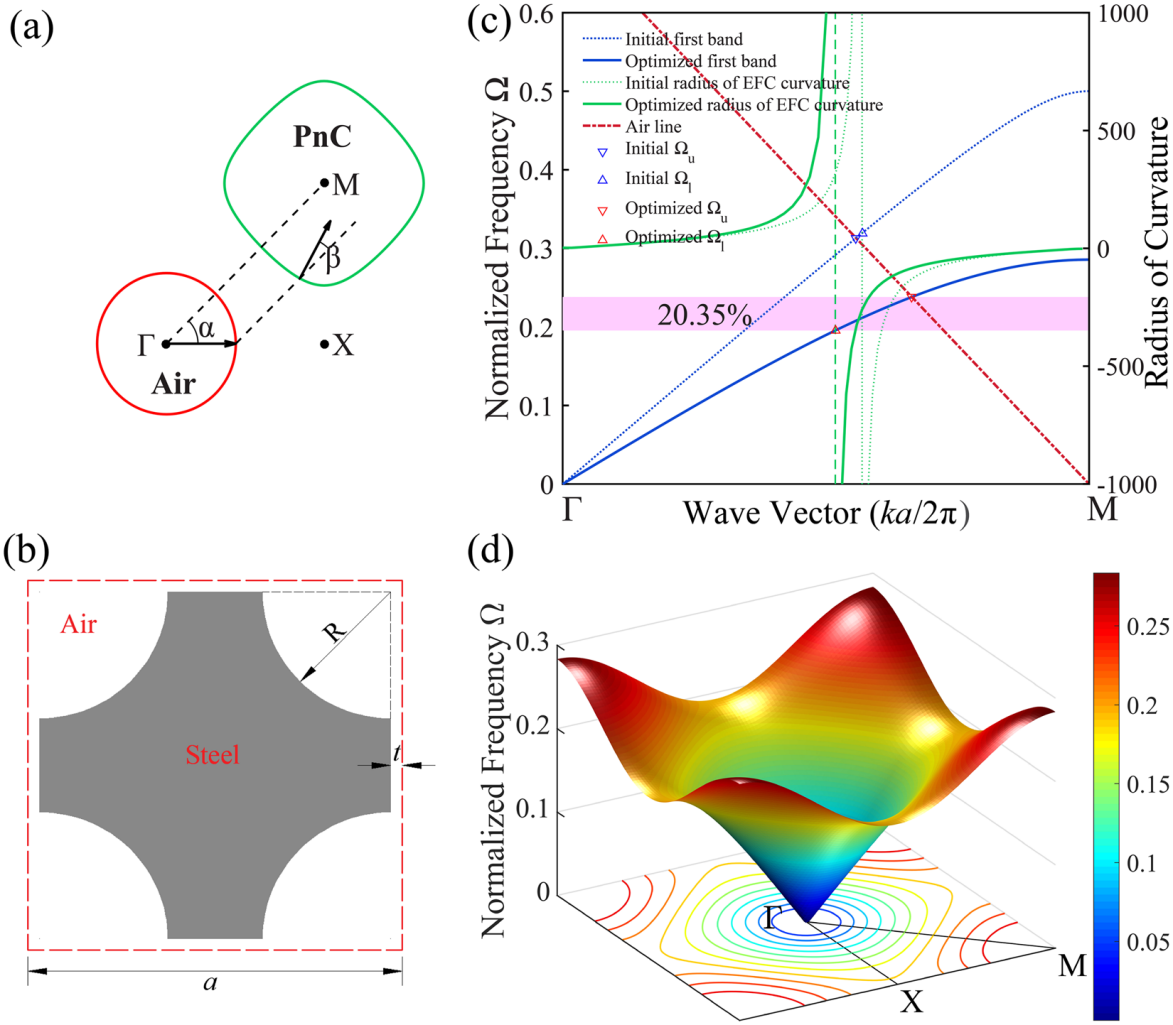
(**a**) Schematic demonstration of negative refraction. Red and green lines denote EFC for air and PnC, respectively. The black arrows indicate directions of energy flow when acoustic waves enter PnC from air with the interface normal parallel to *ΓM*. (**b**) The unit cell of the optimized PnC with square lattice constant *a*. The grey and white denote steel and air respectively. (**c**) The first phononic band of the initial (dotted blue line) and optimized (solid blue line) structure, initial (dotted green line) and optimized (dashed green line) radius of EFC curvature of equifrequency contours along *ΓM* direction and dispersion relation in air which is shifted to *M* point (dashed red line). The initial and optimized upper and lower limit are indicated by blue and red triangles. (**d**) The complete 3D band structure and EFCs of the optimized PnC.

The AANR behavior originates from the relation of equifrequency contours (EFCs) for the PnC and air which defines the lower and upper frequency limits. At the first phononic band, the EFCs for the PnC alter from concave to convex as frequencies get higher in the vicinity of *M* point while the EFCs for air are concentric circles centered at *Γ* point with the radius Ω/*c*_*air*_. For these convex EFCs of the PnC, the group velocity pointing along their outward normal will consequently point inwards to *M* as demonstrated in Fig. 1a. As a result, both the incidence and refraction will stand on the same side of the interface normal, i.e. negative refraction is achieved. Thus the alternation point gives the lower limit of AANR Ω_*l*_. Notice that the group velocity at first band is never opposite to the wave vector, which differs from the LHMs and negative refraction realized in high-order bands. Therefore the realized negative refraction does not come along with either negative index nor backward wave effects. The ultimate case for AANR is that the EFCs for the PnC and air have the same diameter, so as to guarantee negative refraction for incident beams entering PnC from any direction. This condition defines the upper frequency limit, Ω_*u*_ of AANR, and AANR can only be achieved when Ω_*u*_ > Ω_*l*_^9^. Only PnCs with specially designed inclusions can satisfy these strict conditions, which is a typical topology optimization problem.

### Optimized phononic crystal

Our optimization starts from the popular design consisting of steel cylinders in a square lattice. The filling fraction is 50%. Note that there is no AANR behavior found in the initial configuration. BESO gradually updates the topology and enlarges the AANR frequency range as shown in Supplementary Fig. S2a. The final configuration along with its band structure and EFCs are presented in Fig. 1b–d. The results demonstrate that a wide AANR frequency range around Ω = 0.193 ~ 0.237 is obtained, and its relative width, 2(Ω_*u*_ − Ω_*l*_)/(Ω_*u*_ + Ω_*l*_), achieves 20.35%, representing a record value in the literature. The optimized shape of steel has changed significantly from the original circle to a concave curve, and can be approximately characterized by two parameters *R* = 0.34*a* and *t* = 0.03*a*, where *a* is the square lattice constant. The assigned 2*t* air gap between unit cells ensures that only longitudinal waves are supported. The radius of the EFC curvature at the point where EFC and the *ΓM* line intersect is plotted together with the first phononic band and air dispersion line in Fig. 1c. Frequencies Ω are normalized by 2*πc*_*air*_/*a*. The singular point where the radius of EFC curvature goes to positive and negative infinity on two sides gives the lower limit Ω_*l*_ (indicated as upward-pointing triangles), while Ω_*u*_ (indicated as downward-pointing triangles) is given by the intersection of the air line and the first band. Initially, AANR behavior does not exist as Ω_*l*_ > Ω_*u*_ (see blue triangles). With the evolution of the PnC structure, the first phononic band gets depressed, the wave vector corresponding to the Ω_*u*_ gets closer to *M* point. At the same time, the wave vector of Ω_*l*_ approaches *Γ* point. Consequently, the AANR frequency range is enlarged.

**Figure 2:**
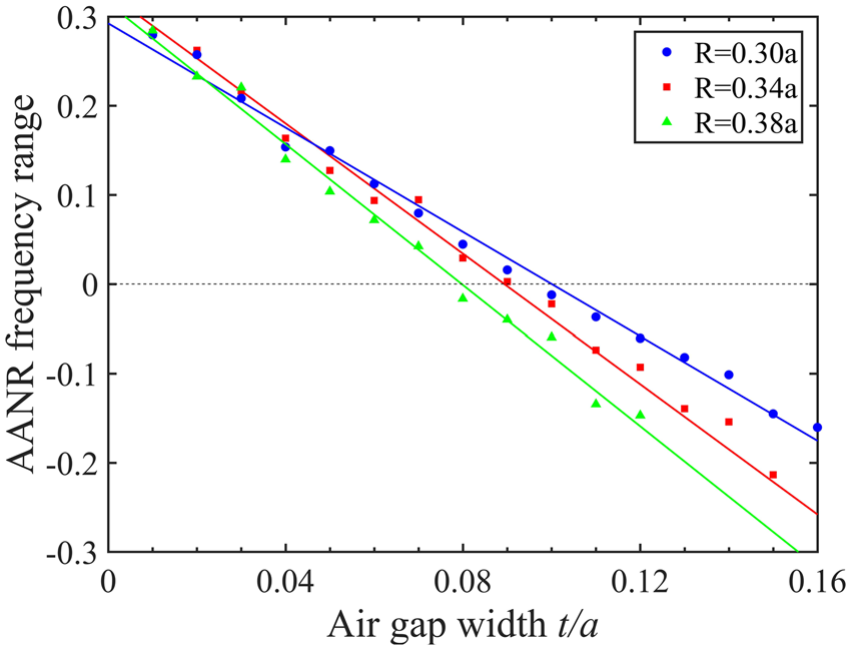
AANR frequency range as a function of air gap with different *R*.

We further investigated the influences of parameters *R* and *t* on AANR for the given topology shown in Fig. 1b. The numerical parametic studies revealed that the assigned air gap 2*t* can substantially tune AANR behavior for a fixed *R*. Figure 2 shows the AANR frequency range as a function of the air gap with different *R*. All the AANR frequency ranges decline monotonically with the increase of the air gap (see the straight fitted lines), and finally disappear. In fact, it is even impossible to obtain AANR by means of topology optimization when the air gap is too wide. In comparison, *R* merely shows a slight influence on AANR when the air gap is fixed. For instance, when *R* increases from 0.1*a* to 0.45*a* along with the fixed air gap 2*t* = 0.625*a*, the AANR behavior persists with its frequency range varying from 9.55% to 20.35%. We envision that the simple cross structure with any thickness would have a positive AANR frequency range as long as the air gap is carefully chosen. This finding deepens our understanding of the relation between the phononic structure and its AANR behavior and provides useful instructions for practical applications.

**Figure 3:**
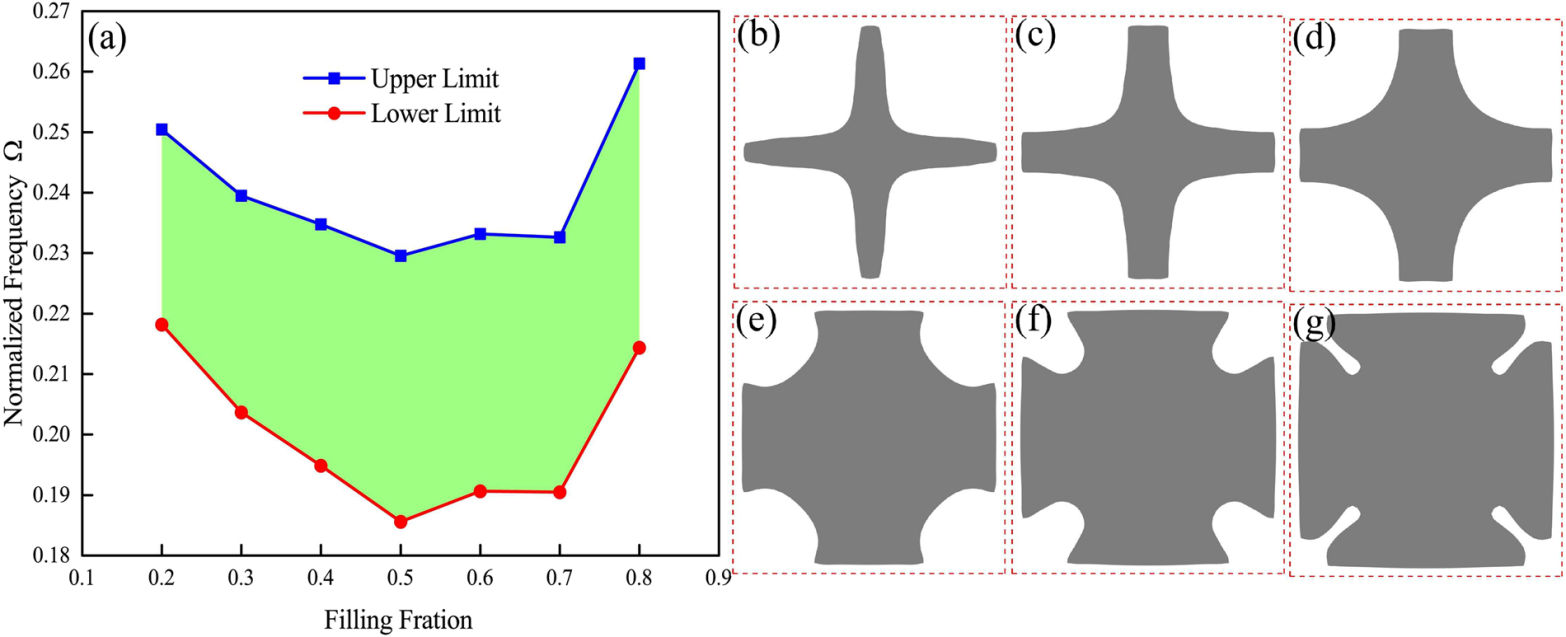
(**a**) Optimized upper and lower limits of AANR frequency range as a function of filling fraction. (**b**–**g**) Optimized unit cells with filling fraction of 20%, 30%, 40%, 60%, 70% and 80%, respectively.

Previous research suggested that AANR does not exist in the initial design at a filling fraction lower than 60%, which means that a high filling fraction has to be employed to realize AANR without tailoring the structure^9^. However, our optimization results indicate that the modification of the geometry can effectively bring about a wide AANR frequency range even at a filling fraction as low as 20% (see Fig. 3a), showing that the AANR is highly dependent on the geometry of PnCs rather than the filling fraction of inclusion. The air gap in all the cases is set to be a constant value 2*t* = 0.625*a*. As the filling fraction decreases from 80% to 20%, both the upper and lower limit first drops and then goes back to a higher level. Among all the cases, the widest AANR is achieved at a filling fraction of 50%. The corresponding optimized unit cells are presented in Fig. 3b–g. It is interestingly observed that all the optimized geometries of inclusion at different filling fractions have concave boundaries. With the decreasing of filling fraction, the optimized topology of inclusions finally evolves to a cross-shaped structure. These concave profiles may contribute to the existence of AANR behavior and need further investigations.

### Validation and application in acoustic focusing

In the following section, we will validate the AANR phenomenon by investigating the refractive properties of the optimized PnC and show that the optimized PnC can be constructed as a multifunctional flat lens which can collimate acoustic waves and focus an acoustic source into an image with a resolution beyond the diffraction limit.

**Figure 4:**
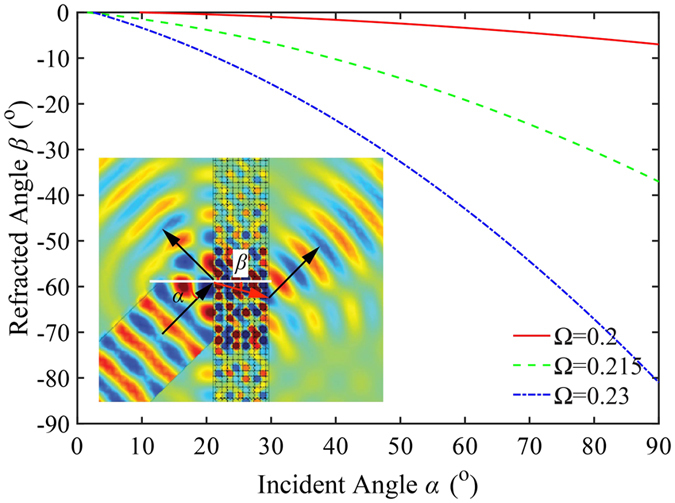
The refracted angle as a function of incident angle at different normalized frequencies. The inset is simulation result for a plane wave through a 10-layer PnC slab with incident angle 45° at frequency Ω = 0.220.

Due to the high anisotropy of EFCs for the optimized PnC, the refracted angle depends on the angle of incidence and frequency, as well as the interface normal. Provided that the interface normal is oriented along *ΓM* direction, the dependence of the refracted angle, *β*, on the incident angle, *α*, at different frequencies is presented in Fig. 4. Negative refracted angles indicate that AANR is achieved in all cases. As the incident angle enlarges from 0° to 90°, the refracted angle changes slightly from 0° to 5.5° for frequency Ω = 0.200 just above the lower limit while it increases greatly from 0° to 64.7° for Ω = 0.230. This phenomenon can be well understood by examining the EFCs of the PnC (see Fig. 1d). The EFC at Ω_*l*_ is close to a square and evolves to circular when frequency approaches Ω_*u*_. The steeper slope of the curve for Ω = 0.230 verifies the fact that EFCs at a higher frequency tends to be more isotropic. The inset of Fig. 4 is the simulation result of a plane wave across a 10-layer PnC slab with an incident angle of 45° at frequency Ω = 0.220, which clearly demonstrates the negative refraction.

**Figure 5:**
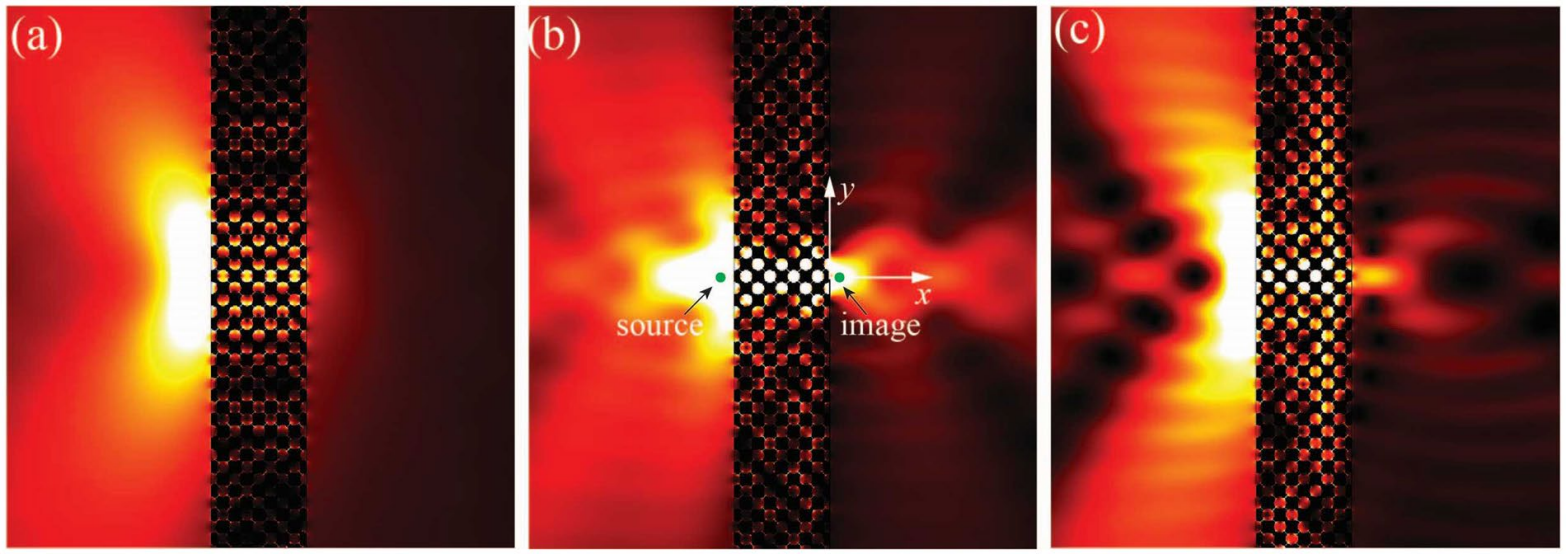
Normalized intensity field of a point source and its image across an 8-layer phononic slab at normalized frequency Ω = 0.200 (**a**), Ω = 0.230 (**b**) and Ω = 0.237 (**c**). The dark (light) color denotes low (high) intensity value.

To check the focusing effect of the optimized PnC, an 8-layer PnC slab consisting of 100 unit cells is constructed with interface normal along *ΓM* direction. The point source is placed at a distance *d* = *a*, close to the left surface of the slab. Absorbing boundary conditions are adopted around the whole simulation domain to avoid reflections. The normalized intensity fields at frequency Ω = 0.200, 0.230 and 0.237 are presented in Fig. 5. For the case of Ω = 0.200, there is no clear image on the right side of the slab. However, a clear guiding channel is visible inside the phononic slab, which demonstrates a self-collimation effect. The reason is that the refracted angles for all incidences are less than 6°. Thus it may need a wider lens to form an evident image as the refracted waves must intersect in the phononic slab (see Supplementary Fig. S3)^3^. Stronger self-collimation effect can be observed as frequency approaches the lower limit Ω = 0.193 due to the extremely flat portion of EFC (see Supplementary Fig. S4).

On the other hand, a clear image with high intensity at frequency Ω = 0.230 has been formed at an approximate distance of 0.6*a* away from the right slab surface. It locates on the axis of the point source without any deviation. The focusing resolution, which is defined as the full width at half maximum of the intensity peak along the *y* direction, is around 0.46*λ*, where *λ* = *a*/Ω is the sound wavelength in air. It reveals that the super resolution beyond the Rayleigh diffraction limit 0.5*λ* has been achieved by the optimized phononic structure. Similarly, a good focusing effect with super resolution 0.37*λ* has been observed at the upper bound of AANR frequency range Ω_*u*_ = 0.237 as well. Such good lensing phenomena benefit from the so-called canalization mechanism^33^. When the EFC of PnC is larger than that of air, a great proportion of evanescent modes of the point source can be canalized to the Bloch modes inside the PnC and transported across the slab without attenuation. All the propagating Bloch modes convert back to the corresponding propagating modes in PnC and air on the exit surface. The substantial canalized evanescent waves attribute to enhance the resolution of focusing. The canalization mechanism is different from the amplification of evanescent waves in the second band and LHMs that are achieved by backwards wave effect introduced phase compensations^13^. As the near circular EFC at higher frequencies of AANR has more uniformly distributed refractive index regardless of the incident direction, the incidences emitted from the point source will be refracted into the same position within the PnC and form a perfect image on the exit side. Meanwhile, the single beam behaviour at frequencies lower than 0.5 × 2*πc*_*air*_/*a*_*s*_ (where ${a}_{s}=\sqrt{2}a$) ensures the high transmission, which is also an important factor for the super resolution.

**Figure 6:**
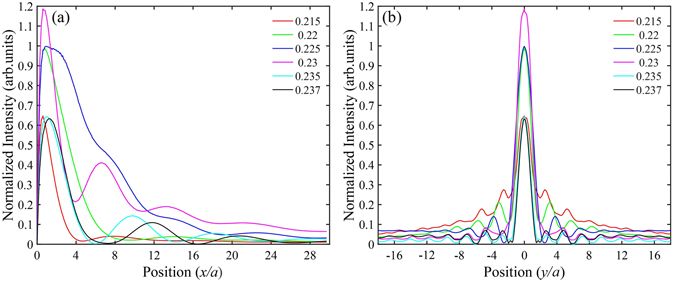
Normalized intensity distributions along the direction perpendicular (**a**) and parallel (**b**) to the phononic slab surface at different frequencies.

It should be pointed out that the thickness and surface termination of the slab can strongly tune the focusing frequency range, which is outside the scope of this letter. As for this 8-layer PnC slab, it can focus a clear spot over frequency range Ω = [0.215,0.237] (see Supplementary Fig. S5). The intensity distributions along the direction perpendicular (*x*) and parallel (*y*) to the slab surface through the peak of focusing point are shown in Fig. 6. Remind that the sound wavelength in air *λ* = *a*/Ω > 4*a* as Ω < 0.25 in all the cases. It is evident that subwavelength imaging has been achieved for these frequencies. In fact the calculated lateral resolution for these frequencies varies from 0.37*λ* to 0.53*λ*. The focal distances are around *a* from the right lens surface, remaining in the near-field domain. These results demonstrate that the optimized PnC enables us to realize self-collimation and subwavelength focusing of acoustic waves simultaneously with a simple flat lens, and has the advantage of working as a superlens over a broad frequency range.

## Conclusion

In summary, we have introduced the topology optimization method for constructing a 2D solid/air PnC displaying AANR phenomenon without negative index. The resulting PnC at a filling fraction 50% exhibits a record AANR frequency range of 20.35% near frequency Ω = 0.215. All the optimized PnCs have concave profiles at material interface which may help to open an effective AANR frequency range. By virtue of the AANR behavior, subwavelength focusing with the super resolution was achieved by a phononic slab near the AANR upper limit while self-collimation took place near the AANR lower limit. The wide frequency range of AANR and subwavelength focusing effect makes the proposed PnC a desirable candidate for the design of acoustic superlens.

## Methods

### Phononic band structure calculation

The 2D PnC considered in this paper is composed of steel inclusion in air background (*ρ*_*steel*_ = 7800 *kg*/*m* ^3^, *ρ*_*air*_ = 1.21 *kg*/*m* ^3^, *c*_*steel*_ = 6100 *m*/*s*, *c*_*air*_ = 334.5 *m*/*s*)^9^, although our approach is general for any type of PnCs. Here, finite element analysis (FEA) is used to calculate the band structure and equifrequency contours since any irregular shape of the inclusion is possible during the optimization process. In the calculation, we ignore the shear modulus of the solid and consider it as metafluid as the solid is nearly rigid in comparison to air^34^. Such assumption was also validated in the simulation of acoustic focusing effect as presented in Fig. S6 in the Supplementary Materials. Numerical simulations are carried out by COMSOL Multiphysics 5.1 throughout this paper.

### Topology optimization formulation

The objective is to find the material distribution of PnCs with the maximal frequency range of AANR, which can be mathematically expressed by

1 $$\begin{array}{rl}\mathrm{Find}: & \quad {\bf{X}}=[{x}_{1},\,{x}_{2},\mathrm{...,}\,{x}_{e},\mathrm{...,}\,{x}_{N}]\quad {x}_{e}\in \mathrm{[0,1]}\\ \mathrm{Maximize}: & \quad f({\bf{X}})={{\rm{\Omega }}}_{u}({\bf{X}})-{{\rm{\Omega }}}_{l}({\bf{X}})\end{array}$$

where *x*_*e*_ (*e* = 1, 2, ..., *N*) is the design variable of element *e*, where *x*_*e*_ = 0 represents element *e* is composed of air and *x*_*e*_ = 1 denotes of steel. *N* is the total number of elements within the primitive unit cell. Thus, the discrete values of design variables can solely represent the material distribution of the unit cell. It should be noted that numerically extracting the lower and upper frequency limits of AANR provides the key for topology optimization.

To establish an optimization algorithm, sensitivity analysis is necessary for evaluating the impact of each element on the frequency range of AANR, which will be used as the optimization criterion. A higher positive value of an elemental sensitivity, i.e. the derivative of the objective function, means that increasing the design variable of the element (physically switching material from air to steel) will increase the AANR frequency range. Following this understanding, BESO will increase design variables (from 0 to 1) for elements with high sensitivities and simultaneously decrease design variables (from 1 to 0) for elements with low sensitivities. As a result, the updated design variables for all elements within the unit cell form a new geometry. The whole optimization procedure including the extraction of the AANR frequency limits, sensitivity analysis, and BESO update scheme is conducted iteratively until the material distribution within the unit cell achieves its optimum. Meanwhile, the AANR frequency range gradually increases from negative, positive to the maximum value. We refer the interested readers to the Supplementary information for the detailed optimization formulation and procedure.

## Electronic supplementary material

Broadband All-Angle Negative Refraction by Phononic Crystals
