# Supplementary material for: Broadband All-angle Negative Refraction by Optimized Phononic Crystals
Source: Sci Rep. 2017 Aug 7;7:7445. doi: 10.1038/s41598-017-07914-1 (PMC5547121; doi:10.1038/s41598-017-07914-1)
Supplement: Supplementary file 1 — Broadband All-Angle Negative Refraction by Phononic Crystals [file 41598_2017_7914_MOESM1_ESM.pdf]

Supplementary Information

Broadband All-Angle Negative Refraction by Phononic Crystals

Yang Fan Li, Fei Meng, Shiwei Zhou, Ming-Hui Lu and Xiaodong Huang

1 Optimization algorithm and process

Before the optimization process, it is important that the upper and lower frequency limits Ω_u and Ω_l are accurately extracted. Taking a PnC consisting of circular steel rods in air with a filling fraction of 50% for example, the variation of radius of the equi-frequency contour (EFC) curvature along ΓM is plotted together with the first phononic band and the air dispersion line in Fig. S1a. As discussed in the manuscript, the point where the sign of radius of EFC curvature alters defines the lower limit Ω_l while the upper limit Ω_u locates at the intersection of the first phononic band and the air line. Fig. S1a shows that AANR does not exist in the current design due to $\Omega_l > \Omega_u$.

Our goal is to find the optimal material distribution of the PnC that possesses a broad all-angle negative refraction (AANR) frequency range at the first band. The optimization objective can be intuitively set to enlarge the difference between the upper and lower limit, i.e. maximizing ($\Omega_u - \Omega_l$). Although Ω_l and Ω_u can be numerically determined, it is quite difficult to mathematically formulate these two values, which causes the challenge in topology optimization. In this paper, we alternatively choose to decrease the lower limit Ω_l by maximizing the radius of curvature at a reference point (its wave vector $|\mathbf{k}|a/2\pi$ = $\left(1-\sqrt{2}/20\right)\Omega_l$) as shown in Fig. S1a. The decrease of Ω_l is equivalent to enlarge the AANR frequency range since Ω_u is determined by the first band and constant air dispersion line. Thus, the optimization problem is defined as

$$\text{Maximize: } R = \frac{\left(f_x'^2 + f_y'^2\right)^{3/2}}{f_{xx}'' \cdot f_y'^2 + f_{yy}'' \cdot f_x'^2 - 2 \cdot f_x' \cdot f_y' \cdot f_{xy}''} \tag{S1}$$

$$\text{Subject to: } V_f = \sum_{e=1}^{N} x_e = V^* \tag{S2}$$

where R is the radius of curvature at the reference point. $f_x' = \partial\Omega/\partial k_x$, $f_y' = \partial\Omega/\partial k_y$. f_{xx}'', f_{yy}'' and f_{xy}'' are the second-order partial derivatives. x_e is the design variable for element e. x_e = 1 denotes element e is solid material while x_e = 0 denotes element e is air; N is the total

number of elements. V_f is the filling fraction of solid material and V^* is the prescribed volume constraint.

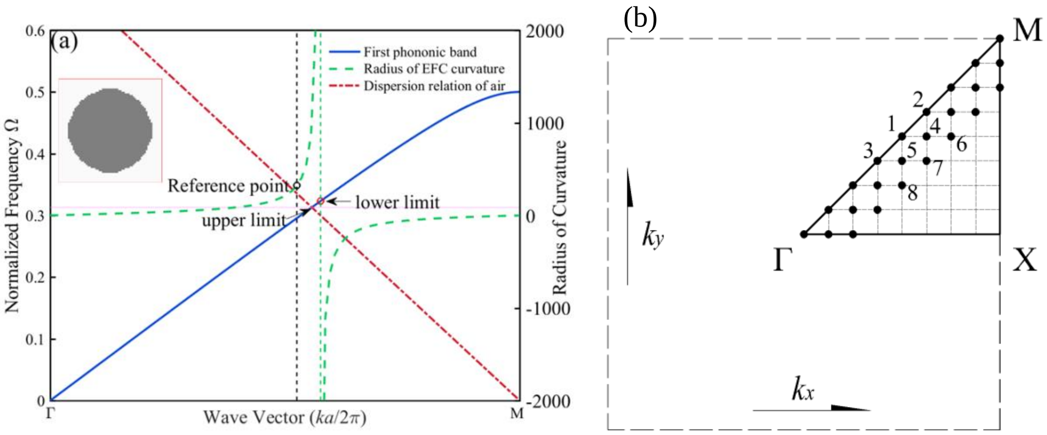

FIG. S1 (a) Band structure of the steel-air phononic crystal (solid blue line) and dispersion relation in air which is shifted to ***M*** point (red dashed line). Frequencies are normalized by $2\pi c_{air}/a$. The reference point is illustrated by a black circle symbol. The inset represents the unit cell. (b) Illustration of square grids within the irreducible Brillouin zone[1]

We consider a square PnC unit cell with C_{4v} symmetry. The band structure and EFCs are calculated using finite element method. The first Brillouin zone is divided into a 100×100 grid, which is illustrated by a coarse grid in Fig. S1b. Take an arbitrary point 1 on the ΓM boundary for example, f'_x , f'_y ,. f''_{xx} , f''_{yy} and f''_{xy} can be expressed by frequencies of neighbor vectors as

$$f'_x = f'_y = \frac{\Omega_4 - \Omega_5}{2 \cdot \Delta k_x} \tag{S3}$$

$$f''_{xx} = f''_{yy} = \frac{\Omega_6 - \Omega_1 + \Omega_8 - \Omega_1}{4 \cdot (\Delta k_x)^2} \text{ and } f''_{xy} = \frac{\Omega_2 - \Omega_7 + \Omega_3 - \Omega_7}{4 \cdot (\Delta k_x)^2} \tag{S4}$$

Therefore, the objective function in Eq. S1 can be further simplified to:

$$\text{Maximize: } R = \frac{\sqrt{2} f'_x}{f''_{xx} - f''_{xy}} \text{ at the reference point} \tag{S5}$$

The sensitivity number α_e for element e can be defined according to the chain rule as

$$\alpha_e = \frac{\partial R}{\partial x_e} = \sum_{i=4,5} \frac{\partial R}{\partial f'_x} \cdot \frac{\partial f'_x}{\partial \Omega_i} \cdot \frac{\partial \Omega_i}{\partial x_e} + \sum_{i=1,6,8} \frac{\partial R}{\partial f''_{xx}} \cdot \frac{\partial f''_{xx}}{\partial \Omega_i} \cdot \frac{\partial \Omega_i}{\partial x_e} + \sum_{i=2,3,7} \frac{\partial R}{\partial f''_{xy}} \cdot \frac{\partial f''_{xy}}{\partial \Omega_i} \cdot \frac{\partial \Omega_i}{\partial x_e} \tag{S6}$$

The derivative of Ω_i with regard to design variable x_e can be calculated by

$$\frac{\partial \Omega_i}{\partial x_e} = \frac{1}{2\Omega_i}\mathbf{u}_i^T\left(\frac{\partial \mathbf{K}}{\partial x_e} - \Omega_i^2\frac{\partial \mathbf{M}}{\partial x_e}\right)\mathbf{u}_i \tag{S7}$$

where $\mathbf{u}_i$ is the eigenvector corresponding to Ω_i. **K** and **M** are elemental stiffness and mass matrix.

A higher positive value of the elemental sensitivity number in Eq. S6 indicates that increasing the design variable of the element (physically switching material from air to steel) will increase the AANR frequency range to a larger extent. In order to maximize R, it is necessary to increase the design variables from $x_e = 0$ to 1 for elements with high sensitivity numbers and decrease the design variables from $x_e = 1$ to 0 for elements with low sensitivity numbers. The design variables are updated according to the following equation.

$$x_e = \begin{cases} 1, \text{ if } \alpha_e > \alpha_{th} \\ 0, \text{ if } \alpha_e < \alpha_{th} \end{cases} \tag{S8}$$

The threshold sensitivity number α_{th} is determined by the volume fraction constraint defined in Eq. S2. Hence, the new phononic structure is formed with updated design variables. This process is repeated until an optimum achieves. More details about BESO can also refer to Huang and Xie[2].

In the following examples, BESO starts from the initial guess shown in Fig. S1a. Figure S2a presents the evolution histories of AANR frequency range and the topology of the phononic unit cell during the optimization process. The filling fraction of solid material is fixed at 50%. It is observed that the AANR frequency range constantly grows and finally stabilizes at a maximum value around 21.19% near its central frequency while the geometry of the unit cell also evolves stably to an optimal. The final optimized structure is surprisingly simple. Except for the preset linear connections, the domain occupied by air can be simplified to four identical quadrants as shown in Fig.1a in the manuscript. The simplified structure in the manuscript, which has an AANR range of 20.35%, only causes a very small discrepancy, indicating the high manufacturing robustness of this design.

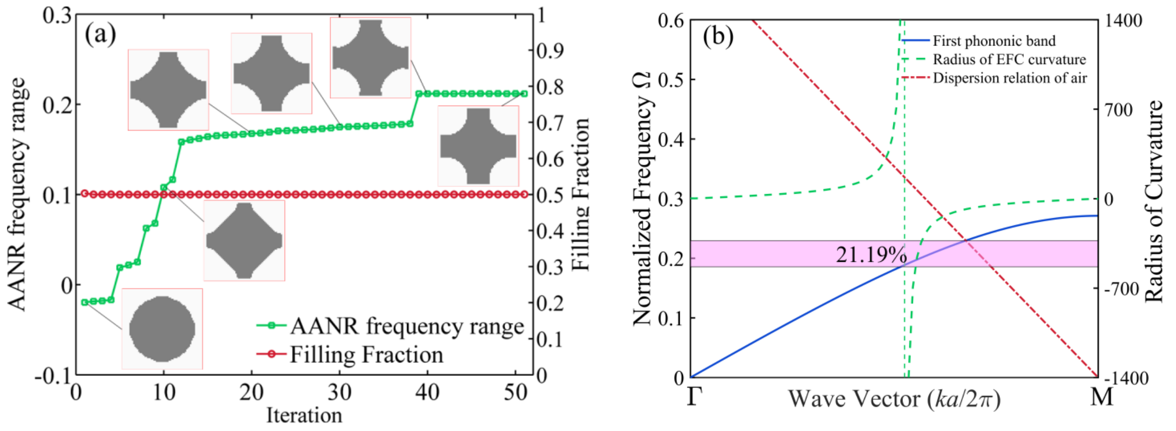

FIG. S2 (a) Evolution histories of AANR frequency range and topology during the optimization at a fix filling fraction of 50%. The white and grey denote air and steel, respectively. (b) Band structure of the optimized phononic crystal and corresponding AANR frequency range.

2 Self-collimation and subwavelength focusing effect of a point source across an eight-layer phononic slab

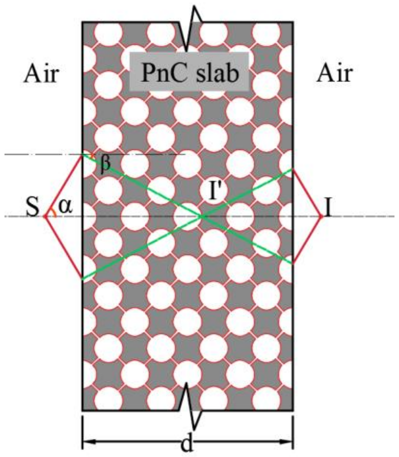

FIG. S3 Schematic illustration of the wave-beam refraction law. When the radiated beams from a point source *S* enter the PnC slab with the incident angle α, they will be negatively refracted and travel along the green lines. For a point source placed on the left side of the flat lens, refractions of radiated waves first meet inside the slab and then meet again and form an image *I* on the right side. Thus, focusing at frequencies that have small refracted angles requires a very wide phononic slab in order to form a clear spot *I* on the right side.

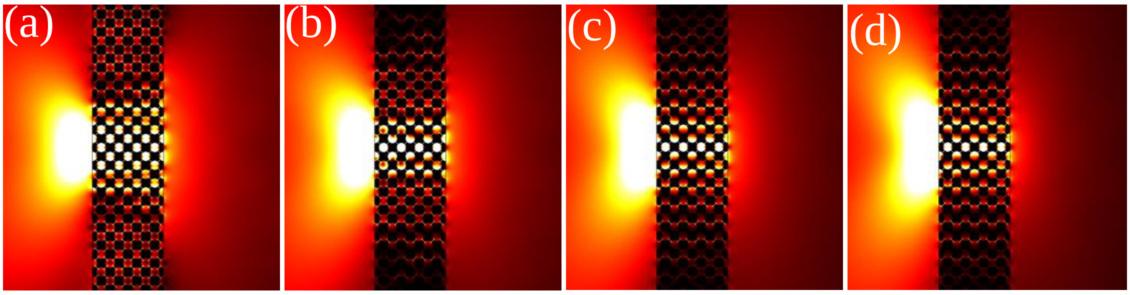

FIG.S4 Normalized intensity field of a point source and its image across an 8-layer phononic slab at a frequency near the lower limit of the AANR range. (a) Ω=0.194, (b) Ω=0.195, (c) Ω=0.196, (d) Ω=0.197. Acoustic waves at these frequencies experience **self-collimation** within the phononic crystal.

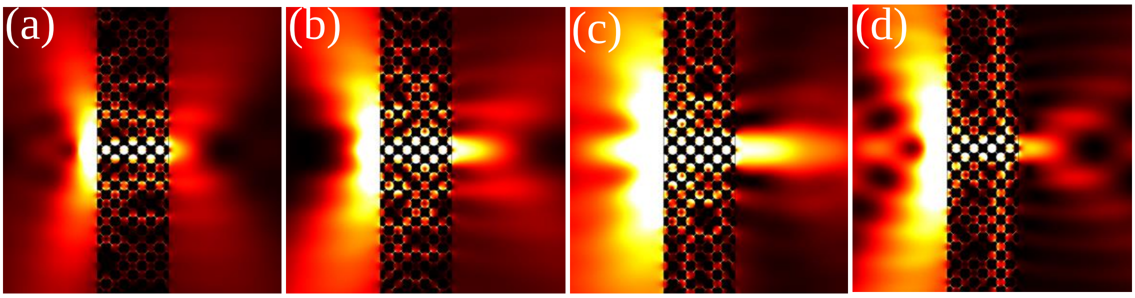

FIG.S5 Normalized intensity field of a point source and its image across an 8-layer phononic slab at a frequency near the upper edge of AANR range Ω=0.215, 0.220, 0.225 and 0.235. Acoustic waves at these frequencies experience **subwavelength focusing** on the right side of the phononic crystal.

3 Validation of metafluid assumption

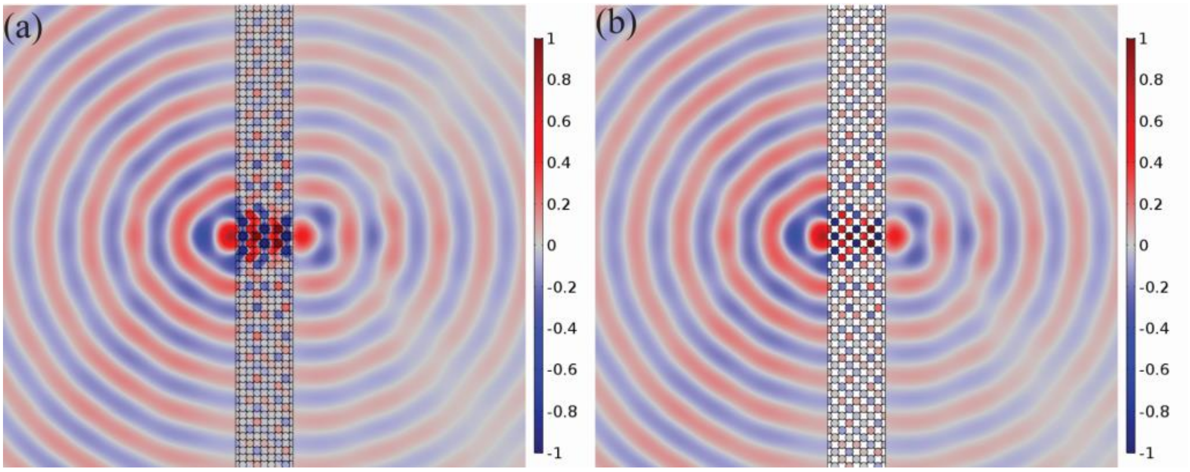

FIG. S6 Simulation results of pressure fields of a point source at the normalized frequency ω=0.23 across an 8-layer slab with (a) and without (b) metafluid assumption. The left model (Fig. 1a) ignores the shear modulus of the solid while the right model treats the air and steel as fluid and solid separately. The identical pressure fields demonstrate the same focusing effect at the normalized frequency Ω=0.23, which indicates that it is suitable to treat the solid as metafluid.

References

1. Fei Meng, Shuo Li, Han Lin, Baohua Jia, and Xiaodong Huang, Finite Elem. Anal. Des. **117–118**, 46 (2016).

2. X. Huang, Y. M. Xie, Evolutionary topology optimization of continuum structures: methods and applications, John Wiley & Sons, Chichester 2010.
